# Monitoring Walking Activity with Wearable Technology in Rural-dwelling Older Adults in Tanzania: A Feasibility Study Nested within a Frailty Prevalence Study

**DOI:** 10.1080/0361073X.2020.1787752

**Published:** 2020-07-08

**Authors:** Silvia Del Din, Emma Grace Lewis, William K. Gray, Harry Collin, John Kissima, Lynn Rochester, Catherine Dotchin, Sarah Urasa, Richard Walker

**Affiliations:** a Translational and Clinical Research Institute, Faculty of Medical Sciences, Clinical Ageing Research Unit, Newcastle University, Newcastle upon Tyne, UK; b Northumbria Healthcare NHS Foundation Trust, North Tyneside General Hospital, North Shields, UK; c Population Health Sciences Institute, Faculty of Medical Sciences, Newcastle University, Newcastle upon Tyne, UK; d The Medical School, Newcastle University, Newcastle upon Tyne, UK; e Hai District Hospital, Boma Ng’ombe, Hai, Kilimanjaro, Tanzania; f Newcastle upon Tyne Hospitals, NHS Foundation Trust, Newcastle upon Tyne, UK; g Kilimanjaro Christian Medical Centre, Moshi, Tanzania

## Abstract

**Background:**

Older adults with lower levels of activity can be at risk of poor health outcomes. Wearable technology has improved the acceptability and objectivity of measuring activity for older adults in high-income countries. Nevertheless, the technology is under-utilized in low-to-middle income countries. The aim was to explore feasibility, acceptability and utility of wearable technology to measure walking activity in rural-dwelling, older Tanzanians.

**Methods:**

A total of 65 participants (73.9 ± 11.2 years), 36 non-frail and 29 frail, were assessed. Free-living data were recorded for 7 days with an accelerometer on the lower back. Data were analyzed via an automatic cloud-based pipeline: volume, pattern and variability of walking were extracted. Acceptability questionnaires were completed. T-tests were used for comparison between the groups.

**Results:**

59/65 datasets were analyzed. Questionnaires indicated that 15/65 (23.0%) experienced some therapeutic benefit from the accelerometer, 15/65 (23.0%) expected diagnostic benefit; 16/65 (24.6%) experienced symptoms while wearing the accelerometer (e.g. itching). Frail adults walked significantly less, had less variable walking patterns, and had a greater proportion of shorter walking bouts compared to the non-frail.

**Conclusion:**

This study suggests that important contextual and practical limitations withstanding wearable technology may be feasible for measuring walking activity in older rural-dwelling adults in low-income settings, identifying those with frailty.

## Introduction

Older adults who have low levels of activity can be at risk of poor health outcomes. However, objectively measuring physical activity can be difficult, with methods such as completion of activity questionnaires or activity diaries subject to recall bias and interpretation bias regarding what constitutes physical activity (Snodgrass et al., 2016). Advancements in wearable technology have improved acceptability, accuracy and objectivity of measuring activity for older adults in high-income countries for discriminate groups (e.g. Parkinson’s disease, stroke, frailty, etc.) and to detect risk (e.g. falls risk) (Althoff et al., 2017; Del Din et al., 2019; Hale, Pal, & Becker, 2008; Lord et al., 2013; Moore et al., 2017). Wearable technology has been extensively used for objectively quantifying and investigating activity in older adults (Alharbi, Straiton, Smith, Neubeck, & Gallagher, 2019; Straiton et al., 2018). Wearable technology for measuring activity includes a plethora of devices (e.g. activPAL™, ActiGraph™, Fitbit™, Axivity AX3, etc.) which may differ in cost, technical specifications (e.g. sampling frequency, battery life, memory storage, dimensions, etc.), type of recorded data (e.g. accelerometry data only, addition of gyroscope data, etc.), availability of raw data (e.g. raw data vs. “epoch-based” data vs. outcomes only), and validity of outcomes (Bassett, Toth, LaMunion, & Crouter, 2017; Evenson, Goto, & Furberg, 2015; Farina & Lowry, 2018; Godfrey et al., 2018). Nevertheless, these technologies have been under-utilized in low- and-middle income countries (LMIC) thus far (Peters et al., 2010).

The physical frailty phenotype (FP), as characterized by Fried et al. (Fried et al., 2001), is identified in relation to five components; unintentional weight loss, low grip strength, self-reported exhaustion, slow walking speed and low physical activity. The presence of three of these characteristics identifies someone as frail. The Minnesota Leisure-time Physical Activity Questionnaire was used in the original paper describing the FP (Fried et al., 2001); however, this is unlikely to be applicable in Sub-Saharan Africa (SSA), where high levels of poverty make physical activity for leisure a luxury, and where older adults are often required to continue to work into their later years, often in manual occupations. Levels of workforce participation for older adults are highest in Africa compared with other world regions and is associated with low levels of pension coverage (UN DESAPD, 2016). The international physical activity questionnaire (IPAQ) and the global physical activity questionnaire have both been used in LMICs, and have attempted to overcome the problem of capturing physical activity data across a variety of cultures and settings (Hallal et al., 2012). Nevertheless, as previously stated, self-reported physical activity data are known to be highly unreliable, and accelerometer data may represent a new “gold standard” for research.

Physical inactivity, in addition to being a component of frailty, is associated with increased mortality (Matthews et al., 2016) and also with age-associated chronic conditions such as obesity and type II diabetes (Lee et al., 2012). Yet physical activity is a modifiable risk factor, and exercise interventions have been shown to improve functioning and reduce the risk of frailty and its adverse outcomes (Clegg, Young, Iliffe, Rikkert, & Rockwood, 2013).

As part of a larger study (Hai District Aging and Frailty Study) (Lewis et al., 2018b), the aim of this work was to explore (i) feasibility, (ii) acceptability, and (iii) utility of utilizing wearable technology and an automatic online pipeline for data transfer and analysis of walking activity for research in a group of rural-dwelling, older Tanzanians.

## Materials and Methods

### Participants

**Figure 1.**
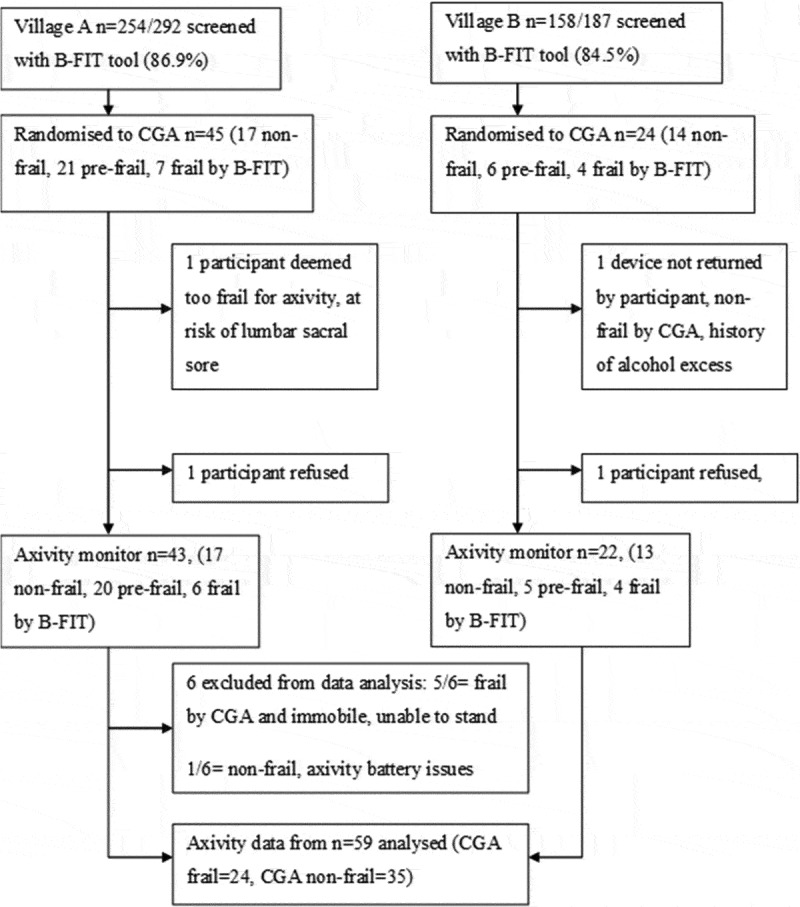
Illustration of the screening and sampling procedure producing data from 59 participants.

This study was part of a larger study investigating frailty in five villages in the Hai District, Northern Tanzania. Methods for this study have previously been described (Lewis et al., 2018b). Individuals were assessed for frailty using the Brief Frailty Instrument for Tanzania (B-FIT) screening tool, this being the first tool for frailty assessment specifically developed for use in a rural low-income country context (Lewis et al., 2018a). Given that this “Axivity feasibility study” was nested within a prevalence study, a random sample of those who were assessed as being pre-frail (50%) and non-frail (10%) by B-FIT was included in the sample, as well as all those screened frail according to B-FIT, as described in detail elsewhere (Lewis et al., 2018b). Thus, a frailty-weighted cohort from two villages was included in the study (Figure 1). Following randomization, 65 participants were assessed by comprehensive geriatric assessment (CGA), 36 were deemed non-frail and 29 frail. CGA frailty assessment was taken as the gold standard for the detection of frailty and was used to validate the B-FIT screen as part of the broader prevalence study (Lewis et al., 2018b).

B-FIT = Brief frailty instrument for Tanzania, CGA = comprehensive geriatric assessment.

Ethics approval was gained from Kilimanjaro Christian Medical Center (KCMC), and the National Institute of Medical Research, ethics committees in Tanzania, and Newcastle University Research Ethics Committee in the UK. All participants were provided with an information sheet in Swahili (the national language) which was read out for those who could not read. All participants signed an informed consent form prior to testing, and those unable to sign provided a thumbprint, or a close relative provided assent (Lewis et al., 2018b).

#### Demographic and Clinical Measures

Demographic data were recorded for each participant. Participants were asked to report their highest educational attainment, whether they could read and/or write, their marital status and whether they currently worked for pay. All questionnaire data were collected by Tanzanian field-workers, who interviewed participants and recorded their responses on ODK to collect open access software using hand-held tablet computers (Android Samsung Galaxy Tab A6).

### Free-living Walking Activity Data Collection: Protocol

**Figure 2.**
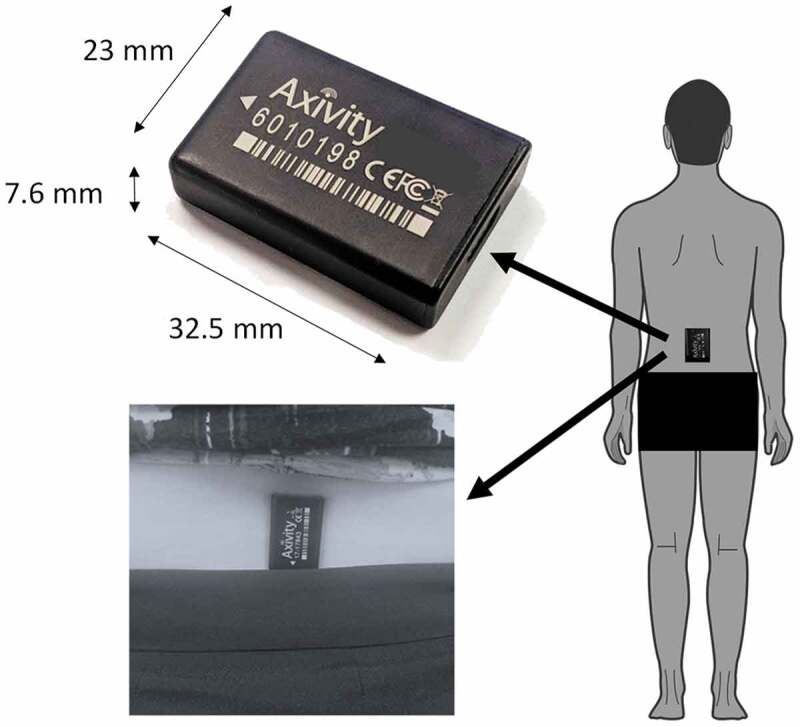
Experimental set up: the Axivity AX3 device, the site of attachment and the orientation of the device on the lower back.

After completing the CGA assessment participants were invited to wear a tri-axial accelerometer-based wearable device (Axivity AX3, York, UK; dimensions: 23.0 × 32.5 × 7.6 mm; weight: 11 g; accuracy of the quartz-stabilized real-time clock: 20 parts per million, battery life: 14 days at 100 Hz, 512Mb flash nonvolatile memory) for 1 week; this device has been validated for suitability in capturing high-resolution data akin to human movement (Del Din, Godfrey, & Rochester, 2016; Ladha, Jackson, Ladha, & Olivier, 2013). The wearable device was located on the fifth lumbar vertebra with a hydrogel adhesive (PAL Technologies, Glasgow, UK) and covered with a Hypafix bandage for extra support (Figure 2). The wearable device was programmed to capture data for 7 days at 100 Hz and at an acceleration range of ± 8 g. Participants were asked to continue their daily activities, including bathing, as usual and not to change their routine. In case the device had to be removed for specific reasons, extra hydrogel adhesives and Hypafix bandage were provided to the participants to re-attach and secure the sensor. An information sheet was given to the participants with the instructions on how to re-attach the sensor. This was also explained verbally and demonstrated to each participant. Upon completion of recording, researchers collected the device in person and completed a verbal feedback questionnaire. The questionnaire comprised several statements, for example, “the device was comfortable to wear” with Likert scale responses to assess the degree to which the statement applied to the participant. Free text responses were also recorded for the questions: “What did you like/dislike about wearing the device?”.

### Data Processing and Analysis

#### Pipeline for Data Processing and Variable Extraction – Free-living Data

**Figure 3.**
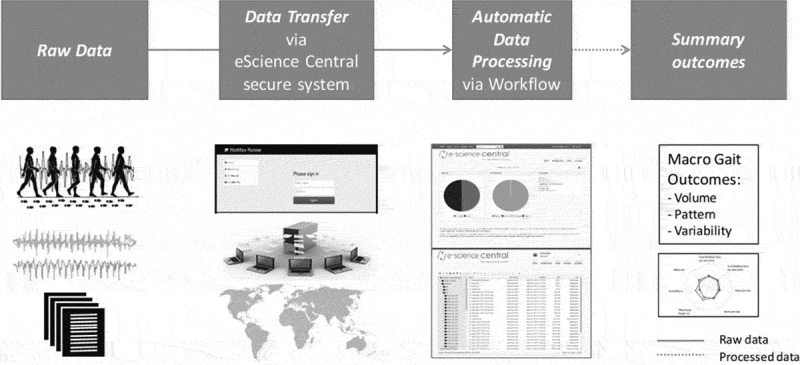
Online analytical platform: framework for analysis and evaluation of free-living outcomes.

Once the wearable device was collected by the researcher, data were uploaded to a secure online platform for automatic data analysis. The pipeline has been developed using e-Science Central (Hiden, Woodman, Watson, & Cala, 2013), a password-protected cloud-based platform that allows the storage, analysis and sharing of data in the cloud (Del Din et al., 2020). Analysis of data was carried out via the e-Science platform using an executable code of validated MATLAB® scripts (Del Din et al., 2019, 2016; Hickey, Del Din, Rochester, & Godfrey, 2017) thereby generating a closed standalone analysis package (Figure 3).

In detail, within the online platform data were segmented (per calendar day). For each day, individual ambulatory bouts (ABs) were extracted, where a ‘bout’ was defined as the continuous length of time spent walking (Del Din, Godfrey, Galna, Lord, & Rochester, 2016). A logical heuristic paradigm was embedded into the walking bout identification and quantification algorithm which has been shown to be accurate in detecting ambulatory bouts (ABs) and step count in free-living conditions. ABs were detected by applying selective thresholds on the standard deviation and the magnitude vector of the tri-axial accelerations (Hickey et al., 2017). All ABs greater than 60 seconds were taken into account for the analysis (Del Din et al., 2019; Weiss et al., 2013; Weiss, Herman, Giladi, & Hausdorff, 2014; Weiss et al., 2011); a threshold of 2.5 seconds was set for the maximum resting period between consecutive ABs (Hickey et al., 2017). We included in the analysis only participants with at least three full days of activity (Bassett et al., 2017).

#### Outcomes

Pooled data were used for quantifying free-living outcomes. Outcomes were described according to a broad framework that captured the overall volume (amount), pattern and variability of walking activity (Del Din et al., 2019, 2020). Volume of walking included total walking time per day, percentage (%) of walking time per day, number of bouts and steps per day. Pattern included mean bout length, generated based on the ABs detected over the 7 days, and a non-linear descriptor (alpha (α)). Alpha describes the distribution of ABs, evaluating the ratio of short to long ABs (i.e. a high alpha means that the total walking time is made up of proportionally short ABs compared to long ABs). AB variability (S_2_) was derived evaluating the ‘within subject’ variability of AB length. Higher AB variability (S_2_) would indicate a more varied walking activity pattern, while lower variability (S_2_) would mean a less varied walking activity, so a reduced engagement in a different activity and a tendency to repeat the same pattern of activity (Lara et al., 2016; Lord et al., 2013).

#### Exposure and Covariates

The primary exposure of interest was frailty which was dichotomized as frail or non-frail based on CGA assessment. In multivariable binary logistic regression modeling, age was used as a covariate. This allowed us to assess the impact of frailty on outcomes having adjusted for age.

#### Statistical Analysis

Statistical analysis was carried out using IBM SPSS (IBM Corp, Armonk, NY, USA). Descriptive and inferential statistics were chosen depending on the nature of the data. Categorical and Likert scale data were summarized using frequencies. Although age data showed some skewness on visual inspection, the mean and standard deviation were reported. To compare the characteristics of frail and non-frail participants, the independent t-test was used for the variable age and Pearson’s Chi-squared test used for all other variables. To compare outcome data from the Axivity monitors (see above) between frail and non-frail participants independent sample t-tests were used for bivariate analysis. Levene’s test was used to assess equality of variance between groups, and t-tests adjusted as appropriate. The data were also investigated with age used as a continuous covariate in multivariable binary logistic regression models. The models are summarized in terms of odds ratios (ORs), 95% confidence intervals (CIs) and tests of significance. Given the exploratory nature of this analysis, we used a threshold of *p* < .05 to guide statistical interpretation.

Free text answers were analyzed by summative content analysis, beginning with word frequency counts that were developed to categorize data into codes (Hsieh & Shannon, 2005). Summative content analysis uses counting to help identify patterns in the data and to contextualize codes by how commonly an idea or meaning is expressed.

## Results

Participants’ baseline clinical and demographic characteristics are presented in Table 1. The frail group was older than the non-frail (*p* < .001). The frailty-weighted sample included proportionally more women (63%) than men. The majority (54%) were not currently married (either widowed, divorced/separated or single), and almost one-third (20/65) 31% had not received a formal education.

**Table 1.** Clinical and demographic characteristics for frail and non-frail participants by CGA.

Characteristic	Frail (n = 29)	Non-frail (n = 36)	Significance
Female (n, %)	22 (75.9%)	19 (52.8%)	X^2^ (1) = 3.67, *p* = .055
Age mean years (SD)	80.3 (12.38)	70.2 (8.41)	**t = 3.89, *p* < .001**
Widowed, separated or single	18 (62.1%)	17 (47.2%)	X^2^ (1) = 1.42, *p* = .233
No education	13 (44.8%)	7 (19.4%)	**X^2^ (1) = 4.85, *p* = .028**
Unable to read/write	16 (55.2%)	7 (19.4%)	**X^2^ (1) = 8.96, *p* = .003**
Not working for pay	26 (89.7%)	21 (58.3%)	**X^2^ (1) = 7.86, *p* = .005**

In bold significant *p*-values (*p* < 0.05).

### Feasibility

The pipeline for data extraction was generally feasible; however, charging the wearable Axivity devices was occasionally hampered by variable access to electricity. Uploading large data files (50,000–260,000 KB) to the cloud was time-consuming owing to slow internet speeds.

Of the 65 datasets uploaded, 59 were analyzed. Data loss (9%) was due to five frail participants who were bedbound and unable to stand, so no height or weight was recorded, and no analysis of walking was possible. One dataset was lost due to sensor battery issues.

### Acceptability

**Figure 4.**
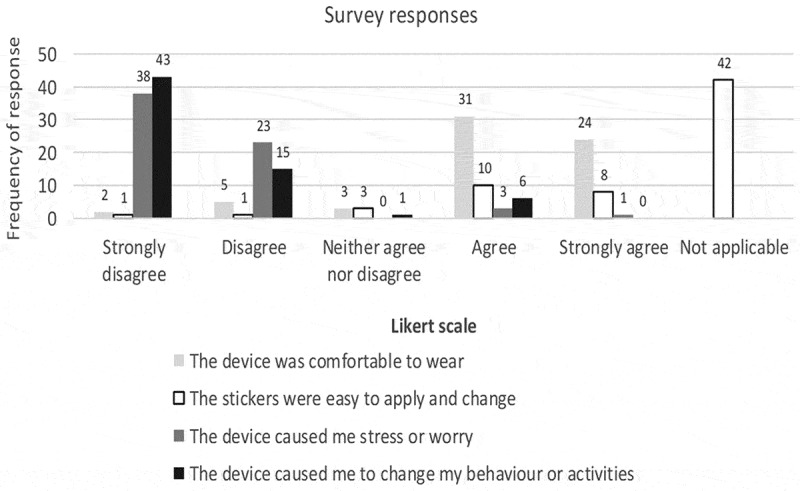
Likert scale responses to statements about the comfort and convenience of the accelerometer devices for n = 65 participants. Note the majority of participants did not change their PALStickies, indicated by answering “not applicable” to the comment “The stickers were easy to apply and change”.

Four main codes were developed through summative content analysis (expectation of therapeutic benefit, expectation of diagnostic benefit, experience of symptoms and worry caused by wearing the device). See Table 2 for full coding. Analysis of participant surveys showed that 15 (23%) of participants reported either experiencing or expecting some therapeutic benefit from wearing the accelerometer. Free text comments analyzed reported apparent improvements in sleep quality, and relief of back and chest pains, for example, one participant stated that the device had “helped her back pain and heaviness.” A further 15 expected some diagnostic benefit, one participant thought “it could show any diseases in his body.” Sixteen (24.6%) experienced symptoms attributed to wearing the accelerometer. The most common was itching, while other symptoms included pain, discomfort and diarrhea. Seven experienced worries about wearing the device. Most commonly these were concerns about dislodging the device during sleep or when washing, but one participant raised concerns that they may be accused of witchcraft if anyone saw the device (Figure 4, Table 2). There was no difference in the experiences of frail compared with non-frail individuals, except that the two participants reporting diarrhea were both frail.

**Table 2.** Feedback questionnaire and examples of free text answers.

What did you like about wearing the device?	Number from n = 65 (%)	Example free text answer
Diagnostic benefit expected	15 (23.0)	He thought this may help diagnose something and improve his wellbeing.
Contributing to the research/trust in researchers	10 (15.3)	She doesn’t understand what it was doing or what it will show but she trusts the healthcare professionals.
Therapeutic benefit expected/experienced	15 (23.0)	She thinks it helped her back pain and heaviness, now feels light.
No inconvenience	4 (6.1)	He could carry on as normal.
No comment	21 (32.3)	-
**What did you dislike about wearing the device?**	**Number from n = 65 (%)**	**Example free text answer**
Experienced symptoms	16 (24.6)	He had some pain from wearing the device when he slept or walked.
Caused worries	7 (10.7)	He was scared people would think he is a witch, if they see the device.
Practical problems applying and reapplying stickers	3 (4.6)	The sticker fell off when washing herself. A 5 year old, helping her couldn’t fix it again.
Didn’t understand purpose	2 (3.0)	She is not sure why we put it there, so we have explained again.
No concerns raised	37 (56.9)	-

### Utility: Differences in Walking Activity between Frail and Non-frail Groups

Results showed that, on average, participants wore the device for 6.60 ± 1.10 days; frail participants wore the device for 6.90 ± 0.44 days and non-frail participants for 6.45 ± 1.35 days. In bivariate analysis (independent sample t-tests), frail participants showed a significantly lower volume of walking (i.e. total walking time per day, percentage of walking time per day, total number of steps and bouts per day), which remained significant when also using age as covariate (Table 3). Frail participants walked in shorter bouts, were less variable and had a greater proportion of shorter walking bouts (higher alpha) compared to the non-frail; these differences were significant in bivariate analysis (independent sample t-tests), but not when accounting for age (Table 3).

**Table 3.** Free-living outcomes for participants grouped as frail and non-frail participants, by CGA. Data are presented for long ambulatory bouts (ABs >60 s). Values are presented as mean (standard deviation).

Outcomes	Frail	Non-frail	Bivariate association (t value, significance)	Multivariable association (odds ratio (95% CI), significance)
**Volume**				
Total Walking Time per Day (Minutes)	14.036 (18.567)	99.509 (66.413)	**6.977, *p* < .001**	**0.939 (0.904 to 0.975), *p* < .001**
Percentage of Walking Time	0.975 (1.289)	6.909 (4.612)	**6.975, *p* < .001**	**0.401 (0.232 to 0.693), *p* < .001**
Number of steps per Day	1029 (1432.725)	8409.182 (5916.353)	**6.857, *p* < .001**	**0.999 (0.999 to 1.000), *p* = .001**
Bouts per Day	5.803 (6.958)	35.879 (18.408)	**8.482, *p* < .001**	**0.835 (0.753 to 0.926), *p* < .001**
**Variability**				
Variability (S_2_)	0.430 (0.258)	0.583 (0.155)	**2.413, *p* = .023**	0.049 (0.002 to 1.171), *p* = .062
**Pattern**				
Mean Bout Length (Seconds)	124.578 (58.848)	157.428 (49.13)	**2.217, *p* = .031**	0.990 (0.978 to 1.002), *p* = .091
Alpha (α)	4.457 (3.72)	2.693 (0.734)	**2.097, *p* = .049**	1.754 (0.876 to 3.512), *p* = .112

Significant *p*-values (*p* < 0.05) are presented in bold.

## Discussion

This feasibility study is encouraging in terms of using wearable technology for research in rural-dwelling older adults in LMICs: we showed that despite some technical and practical issues, the wearable sensor was tolerated by the majority of the participants, although important contextual differences influenced participants’ understanding of the technology. Preliminary results showed also that macro gait outcomes look promising in discriminating between frail and non-frail older adults.

### Feasibility

This study was successful in large part due to the research team’s close relationship with the village enumerators who were involved in recruiting participants to screening. It was feasible to deploy the wearable technology; however, it was a labor-intensive process, with repeated home visits required at 1 week apart in order to collect the devices, as the postal system was not reliable or rapid enough. Uploading data files was also a challenging and time-consuming process due to large file sizes and slow, unreliable internet connections. Due to participants not having ready access to affordable electricity, the research team took responsibility for storing and charging the devices.

Future work should investigate perceptions of technology (e.g. through focus groups) before utilizing a specific device, in order to understand the expectations and acceptability of this type of technology in LMIC study settings (Godfrey et al., 2020). A lack of access to quality affordable health care as well as low levels of literacy may have influenced the perceptions of participants in this study. Despite low numbers admitting that they did not fully understand the aims of the study, qualitative analysis of the participant surveys suggests a mismatch of perceptions and expectations from participating. It would be interesting to explore the use of wearable technology for screening during hospital clinic visits in this setting, in order to investigate the link between in-clinic mobility and frailty-related clinical outcomes. This could limit the labor-intensive process of free-living screening.

### Acceptability

An important minority (23%) expressed expectations of therapeutic and diagnostic benefit, and 24.6% described symptoms attributed to wearing the accelerometer, however they were otherwise well tolerated. An accelerometer pilot study in rural Ghana similarly found that “a few” participants thought the devices were a form of “medical aid” that could help them to work more productively on the farm (Zanello, Srinivasan, & Nkegbe, 2017). Notably, itching at the sticker site was the most commonly reported adverse symptom, yet 42 (65%) did not change the sticker as advised. It is unclear why so many participants chose not to re-apply the sticker, despite this having been demonstrated and explained during the consenting process. Only three participants described problems re-applying the stickers, all of whom were non-frail. It can be concluded that this type of technology can be used in research in rural LMIC environments. However, in the absence of adequate primary health care it is understandable that participants hoped to receive some benefit from participating, as would be the case from an interventional or diagnostic study. In the context of poor access to health care low education levels, technology for global health research should be carefully utilized with these understandable misperceptions in mind.

### Utility: Differences in Walking Activity between Groups

An important limitation in the use of this wearable technology for the assessment of frail individuals is the fact that immobile and bed-bound frail participants were excluded from these data analyses (given that this was an analysis of walking). Wearable technology which estimates caloric expenditure from activities other than walking may permit analysis of the physical activity of the frailest individuals. In agreement with previous work showing that frailty and functional impairment are associated with low physical activity in older adults, as measured by accelerometry technology (Chigateri, Kerse, Wheeler, MacDonald, & Klenk, 2018; Clarke et al., 2017; Huisingh-Scheetz et al., 2018), we found that frail participants were not as active as non-frail and had a significantly lower volume of walking activity. This aspect was found to be particularly influenced by frailty beyond just normal aging.

Differences were also observed in the pattern and variability of all walking bouts. Frail individuals tended to have a greater number of shorter walking bouts (higher alpha), a lower bout length and a lower variability in walking bout duration with respect to non-frail older adults. The combined information of higher alpha, lower variability (S_2_), and lower mean bout length gives a better picture of the walking pattern of frail older adults who appear to walk with a greater proportion of short bouts and with a low variability, possibly suggesting that frail older adults may not be able to engage in a variety of different walking activities (including short and long bouts), may be restricted to activities entailing short walking bouts (e.g. within the home environment) and may be unable to sustain prolonged bouts of walking.

Combining accelerometry data with time-use data would provide a better understanding of the rural agricultural and livelihood activities that older adults living with and without frailty undertake in this setting (Zanello et al., 2017). While this pilot study has succeeded in showing differences between frail and non-frail older adults’ free-living walking patterns, future work should seek to validate these data against self-reported surveys such as the international physical activity questionnaire (IPAQ) (Oyeyemi, Umar, Oguche, Aliyu, & Oyeyemi, 2014), or time-use questionnaires which would be more practical outside of the research context for investigating physical activity levels in the context of frailty. Future work should also consider validating walking activity detection methods in rural-dwelling populations: although the methods used for the identification of walking bouts and step counts were validated in high-income countries, these methods may have some limitations when used in rural-dwelling populations in LMICs.

## Conclusion

This pilot study is the first study known to authors that compares accelerometry data by CGA-diagnosed frailty in a sample of rural-dwelling older adults in SSA. This work showed encouraging data suggesting that wearable technology may be a feasible method for accurately measuring activity levels in older rural-dwelling adults in low-income settings. The findings confirm that frailty was associated with a reduced volume of walking, shorter walking bouts and reduced variability in walking bouts. While this pilot study was successful, for the scale-up of future research projects, common misperceptions that the accelerometry device may provide diagnoses or therapeutic benefit to individual participants should be carefully countered, and the time and personnel-intensive nature of conducting this work should be noted. Future work might investigate how walking patterns differ between rural and urban settings and could attempt to estimate older people’s energy expenditure for more accurate characterization of frailty in the rural SSA setting.
